# Diagnostic Values of Laboratory Biomarkers in Predicting a Severe Course of COVID-19 on Hospital Admission

**DOI:** 10.1155/2022/5644956

**Published:** 2022-11-07

**Authors:** Anusha Tummala, Venkat Ramesh, Nagalla Balakrishna, Rajeswari Koyyada, Anula Divyash Singh, Sreekanth Patnam, M. Satish Kumar, Sneha Varahala, Sasidhar V. Manda, Suneetha Narreddy

**Affiliations:** ^1^Apollo Hospitals Educational and Research Foundation (AHERF), Cell and Molecular Biology Research Lab, 500033, Hyderabad, India; ^2^Department of Infectious Diseases, Apollo Health City, Jubilee Hills, Hyderabad 500033, India; ^3^Department of Biostatistics, Apollo Institute of Medical Science and Research (AIMSR), Jubilee Hills, Hyderabad 500033, India; ^4^IIT Hyderabad, Department of Biomedical Engineering, 502285, Kandi, India; ^5^Blood Bank and Transfusion Unit, Apollo Health City, Jubilee Hills, 500033, Hyderabad, India

## Abstract

**Objective:**

We intend to identify differences in the clinicodemographic and laboratory findings of COVID-19 patients to predict disease severity and outcome on admission.

**Methods:**

This single-centred retrospective study retrieved laboratory and clinical data from 350 COVID-19 patients on admission, represented as frequency tables. A multivariate regression model was used to assess the statistically significant association between the explanatory variables and COVID-19 infection outcomes, where adjusted odds ratio (AOR), *p* value, and 95% CI were used for testing significance.

**Results:**

Among the 350 COVID-19 patients studied, there was a significant increase in the WBC count, neutrophils, aggregate index of systemic inflammation (AISI), neutrophil-to-lymphocyte ratio (dNLR), neutrophil-to-lymphocyte and platelet ratio (NLPR), monocyte-to-lymphocyte ratio (MLR), systemic immune-inflammation index (SII), systemic inflammation response index (SIRI), D-dimer, interleukin-6 (IL-6), ferritin, lactate dehydrogenase (LDH), prothrombin time (PT), glucose, urea, urea nitrogen, creatinine, alanine phosphatase (ALP), and aspartate aminotransferase (AST) and a significant decrease in lymphocytes, eosinophils, total protein, albumin, prealbumin serum, and albumin/globulin (A/G) ratio in the severe group when compared with the mild and moderate groups. However, after adjusting their age, gender, and comorbidities, WBC count (adjusted odds ratio (AOR) = 6.888, 95% CI = 1.590-29.839, *p* = 0.010), neutrophils (AOR = 5.912, 95% CI = 2.131-16.402, *p* = 0.001), and urea (AOR = 4.843, 95% CI = 1.988-11.755, *p* = 0.001) were strongly associated with disease severity. *Interpretation and Conclusion*. On admission, WBC count, neutrophils, and urea, with their cut of values, can identify at-risk COVID-19 patients who could develop severe COVID-19.

## 1. Introduction

The novel coronavirus (COVID-19) was declared a global pandemic by the World Health Organization (WHO) on March 11, 2020 [1]. COVID-19 exhibits heterogeneous disease patterns due to variable clinical factors, including age, gender, comorbidities, genetic makeup, and acquired immune differences [2]. Therefore, clinical assessment of COVID-19 is essential for effective management of the infection and its clinical course. Furthermore, COVID-19 is a multisystemic disease regulated by an interplay of immunological, inflammatory, and coagulative cascades [3]. Therefore, biomarker panels could provide reliable information for effective clinical decision-making [3, 4].

As there is no targeted therapy for COVID-19, identifying laboratory biomarkers that could predict the severity of the disease at an early stage would be helpful in monitoring disease progression [5]. Through complete blood picture analysis, abnormal cell counts, like leukopenia, increased neutrophil count, and decreased platelet count, are associated with severe disease and worse outcomes in hospitalized patients [6, 7]. Also, due to the cytokine storm, there is an increase in interleukins, C-reactive proteins, and coagulation markers in severe COVID-19 patients [8, 9]. However, limited studies assessed the role of laboratory markers in predicting disease severity in the Indian population.

The objectives of this study were to evaluate the baseline characteristics and identify the predictive value of the above markers for stratifying disease severity using univariate and multivariate regression analysis approaches.

## 2. Material and Methods

### 2.1. Study Design and Setting

The study is a single-centred retrospective study, and the patients are randomly recruited and have completed data sets during the period March–September 2020. The recruitment coincided with the first wave in India. About 350 patients were retrieved from Apollo Hospital, Hyderabad. The institutional ethical committee approved this study and has waived the need for informed consent. This study included laboratory-confirmed COVID-19 adult patients (≥18 years). RT-PCR method was used as the gold standard to confirm the COVID-19 status. Patients were categorized as mild, moderate, and severe based on the clinical guidelines by AIIMS/ICMR COVID-19 National Task Force/joint monitoring group.

- Mild: characterized by upper respiratory infection, fever, cough, sore throat, or difficulty breathing
- Moderate: patients with lower respiratory tract infections based on the respiratory rate ≥ 24/min and Spo2 levels ≤ 93% on room air
- Severe: patients with respiratory rate > 30/min, breathlessness, and Spo2 levels < 90% on room air. These patients mainly required oxygen

### 2.2. Data Collection

All vital parameters, including pulse, blood pressure, respiratory rate, and peripheral capillary oxygen saturation (Spo2), were recorded immediately upon admission.

All biochemical procedures were performed on the first day of hospital admission. Complete blood cell count (CBC) and biochemical parameters such as interleukin-6 (IL-6), glucose, creatinine, urea, cholesterol, total protein (TP), albumin, globulin, A/G ratio, aspartate aminotransferase (AST), alanine aminotransferase (ALT), alkaline phosphatase (ALP), gamma-glutamyl transferase (GGT), lactate dehydrogenase (LDH), C-reactive protein (CRP), prothrombin time (PT), activated prothrombin time (APTT), and D-dimer were analysed.

### 2.3. Blood Cell Derived Inflammation Markers

Previous studies have shown that severe COVID-19 patients had increased neutrophil and monocyte counts and decreased lymphocyte and platelet levels. Moreover, combined ratios of these parameters are used as inflammation indexes and biomarkers to assist in diagnosing and prognosis of inflammation diseases [10].  Table 1 represents the formulas to calculate these indices.

### 2.4. Data Handling and Management

Categorical variables have been represented as percentages in demographic distribution. In continuous variables, pulse, respiratory rate, and Spo2 have been denoted as mean ± standard deviation, whereas age and gender are expressed as percentages. A chi-squared test was used to determine the association between disease severity and baseline laboratory markers in the cross-tabulation method. Continuous variables were converted into categorical variables according to their reference values. *p* < 0.05 was considered statistically significant. We further carried out multivariate logistic regression analysis to exclude the insignificant variables in the cross-tabulation model. Finally, the relative risk was assessed using ninety-five percent confidence intervals (95% CI) of odds ratio (OR) and adjusted odds ratio (AOR) against age, gender, and comorbidities to find the association between biomarkers and the severity outcome. ROC curve analysis was done to predict the diagnostic efficiency between biomarkers and the severity outcome.

## 3. Results

### 3.1. Demographic, Clinical Characteristics, and Presenting Symptoms

Table 2 represents the demographic and clinical characteristics of COVID-19 patients. Among the 350 patients, 117 were categorized as mild, 99 as moderate, and 134 as severe upon admission. Among them, 76 were nonsurvivors, and the remaining 274 were survivors. There was a significant increase in patients aged > 60 years (53.9%) in the severe category when compared with the mild (15.6%) and moderate groups (30.5%). In addition, an increased percentage of younger patients (18-44 years) were seen in the mild (64.3%) group when compared to the moderate (23.5%) and severe (12.2%) groups. Further, the frequency of male patients is significantly (*p* < 0.001) higher than female patients in this study.

Compared to mild and moderate patients, patients with comorbidities were higher in the severe group. Among them, the frequency of diabetes mellitus was higher in the severe (51.6%) and moderate (33.3%) groups when compared with the mild (15.1%) group. Hypertension patients were seen in the severe (51.3%) and moderate (32.7%) groups when compared with the mild (16%) group. Coronary artery disease (CAD) was higher in the severe (55.2%) and moderate (36.2%) when compared with the mild (8.6%) group. Chronic kidney disease (CKD) was seen only in the severe (76.9%) and moderate (23.1%) groups. Other comorbidities observed were the chronic disease of the lung, liver, and neurological disorders. They constitute 42.1% in severe, 30.5% in moderate, and 27.4% in the mild groups.

Symptom-wise, 35.2% of patients incurred fever in the severe group compared to 30% in the moderate group and 34.8% in the mild group. 37% of participants had a dry cough in the severe group, whereas 30% and 33.3% were moderate and mild. No significant differences were observed for fever and dry cough among the groups. Sore throat was observed in 57.5% of the mild category, 37.5% of the moderate category, and 5% of the severe group. Difficulty in breathing was seen in 58.2% of the severe group and 27.4% of the moderate group when compared to 14.4% of the mild group.

On admission, there is an increase in pulse and respiratory rate in the severe (98.14 ± 23.11; 27.98 ± 7.8) group when compared with the moderate (87.07 ± 12.75; 22.78 ± 4.3) and mild (86.94 ± 13.74; 21.31 ± 6.039) groups. In addition, Spo2 levels were decreased in the severe group (85.6 ± 16.84) when compared with the moderate (95.96 ± 3.715) and mild (97.42 ± 2.207) groups.

### 3.2. Baseline Hematological Biomarker Levels

**Figure 1:**
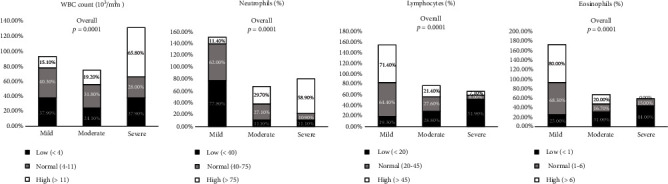
Comparison of baseline hematological parameters between mild, moderate, and severe groups of COVID-19 patients on admission. Data are represented as *n* (%).

Figure 1 (Supplementary Table S1) represents the frequency percentage of haematological parameters on admission based on COVID-19 severity. WBC count showed a significant (*p* > 0.0001) increase in the severe category compared with the mild and moderate groups among the haematological parameters. Neutrophils were also significantly increased (*p* < 0.0001) in the severe, mild, and moderate groups. Neutrophil percentage in the range of <40, 40-75, and >75 was 11.1%, 10.9%, and 58.9% in the severe group, whereas 11.1%, 27.1%, and 29.7% in the moderate group and 77.8%, 62.0%, and 11.4% in the mild group. Lymphocytopenia was observed in the severe group (*p* < 0.0001) compared with the moderate and mild groups. Eosinophil count showed a significant (*p* < 0.0001) decrease in the severe group compared to the mild and moderate groups. The percentage of eosinophils in the range of <1, 1-6, and >6 was 44.0%, 15.0%, and 0% in the severe group, whereas 31.0%, 16.7%, and 20.0% in the moderate group and 25.0%, 68.3%, and 80.0% in the mild group.

### 3.3. Blood Cell Count Inflammation Index Markers

**Figure 2:**
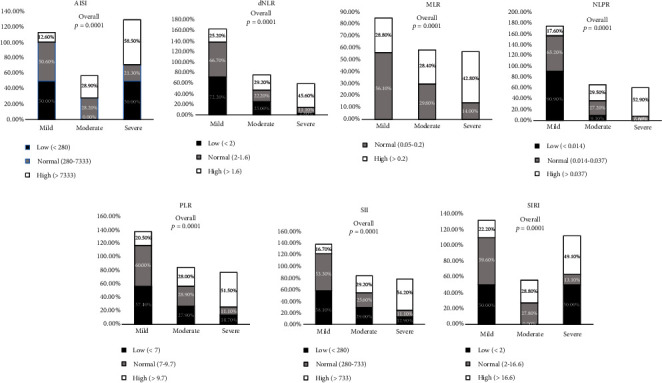
Comparison of baseline blood cell count derived inflammation index markers between mild, moderate, and severe groups of COVID-19 patients on admission. Data are represented as *n* (%).

Figure 2 (Supplementary Table S2) represents the frequency of inflammation index levels of COVID-19 severity on admission.

Among the inflammation indices, except for MPR, all other ratios showed a significant (*p* < 0.0001) increase in the severe group compared to the moderate and mild groups. The percentage of AISI in the range of <280, 280-7333, and <7333 was 50.0%, 21.3%, and 58.5% in the severe group, whereas 0%, 28.2%, and 28.9% in the moderate group and 50.0%, 50.6%, and 12.6% in the mild group. The percentage of dNLR in the range of <2, 2-1.6, and >1.6 was 2.8%, 11.1%, and 45.6% in the severe group, whereas 25.0%, 22.2%, and 29.2% in the moderate group and 72.2%, 66.7%, and 25.2% in the mild group. The percentage of MLR in the range of 0.05-0.2 and >0.2 was 14.0% and 42.8% in the severe group, whereas 29.8% and 28.4% in the moderate group and 56.1% and 28.8% in the mild group. The percentage of NLPR was 0%, 7.6%, and 52.9% in the severe group, whereas 9.1%, 27.2%, and 29.5% in the moderate group and 90.9%, 65.2%, and 17.6% in the mild group. The percentage of PLR in the range of <7, 7-9.7, and >9.7 was 14.7%, 11.1%, and 51.5% in the severe group, whereas 27.9%, 28.9%, and 28.0% in the moderate group and 57.4%, 60.0%, and 20.5% in the mild group. The percentage of SII in the range of <280, 280-733, and >733 was 12.9%, 11.1%, and 54.2% in the severe group, whereas 29.0%, 25.6%, and 29.2% in the moderate group and 58.1%, 63.3%, and 16.7% in the mild group. The percentage of SIRI in the range of <2, 2-16.6, and >16.6 was 50.0%, 13.1%, and 49.1% in the severe group, whereas 0%, 27.3%, and 28.8% in the moderate group and 50.0%, 59.6%, and 22.2% in the mild group.

### 3.4. Inflammation and Coagulation Marker Levels on Admission

**Figure 3:**
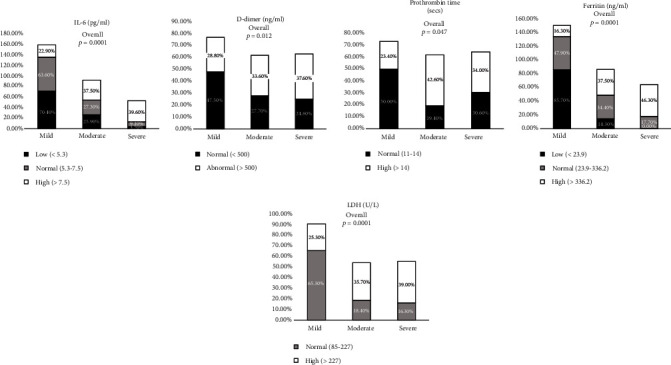
Comparison of baseline inflammation and coagulation marker levels between mild, moderate, and severe groups of COVID-19 patients on admission. Data are represented as *n* (%).

Figure 3 (Supplementary Table S3) represents the frequency percentage of inflammation and coagulation marker levels on admission regarding COVID-19 severity. IL-6 (*p* < 0.0001), D-dimer (*p* < 0.012), PT (<0.047), ferritin (<0.0001), and LDH (<0.0001) showed a significant increase in the severe group when compared with the mild and moderate groups. The percentage of IL-6 in the range of <5.3, 5.3-7.5, and >7.5 was 3.7%, 9.1%, and 39.6% in the severe group, whereas 25.9%, 27.3%, and 37.5% in the moderate group and 70.4%, 63.6%, and 22.9% in the mild group. The percentage of D-dimer in the range of <500 and >500 was 24.8% and 37.6% in the severe group, whereas 27.7% and 33.6% in the moderate group and 47.5% and 28.8% in the mild group. The percentage of PT in the range of 11-14 and >14 was 30.6% and 34.0% in the severe group, whereas 19.4% and 42.6% in the moderate group and 50.0% and 23.4% in the mild group. The percentage of ferritin in the range of <23.9, 23.9-336.2, and >336.2 was 0%, 17.7%, and 46.3% in the severe group, whereas 14.3%, 34.4%, and 37.5% in the moderate group and 85.7%, 47.9%, and 16.3% in the mild group. The percentage of LDH in the range of 85-227 and >227 was 16.3% and 39.0% in the severe group, whereas 18.4% and 35.7% in the moderate group and 65.3% and 25.3% in the mild group.

### 3.5. Liver Function Test (LFT) Marker Levels on Admission

**Figure 4:**
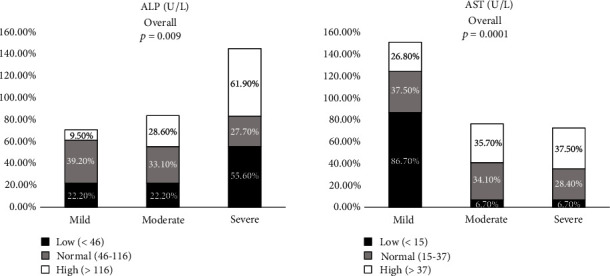
Comparison of baseline liver function test (LFT) marker levels between mild, moderate, and severe groups of COVID-19 patients on admission. Data are represented as *n* (%).

Figure 4 (Supplementary Table S4) represents the frequency percentage of LFT marker levels on admission in COVID-19 patients. ALP (*p* < 0.009) and AST (*p* < 0.001) showed a significant increase in the severe group when compared with the mild and moderate groups. The percentage of ALP in the range of <46, 46-116, and >116 was 55.6%, 27.7%, and 61.9% in the severe group, whereas 22.2%, 33.1%, and 28.6% in the moderate group and 22.2%, 39.2%, and 9.5% in the mild group. The percentage of AST in the range of <15, 15-37, and >37 was 6.7%, 28.4%, and 37.5% in the severe group, whereas 6.7%, 34.1%, and 35.7% in the moderate group and 86.7%, 37.5%, and 26.8% in the mild group.

### 3.6. Renal Function Test and Other Biomarker Levels on Admission

**Figure 5:**
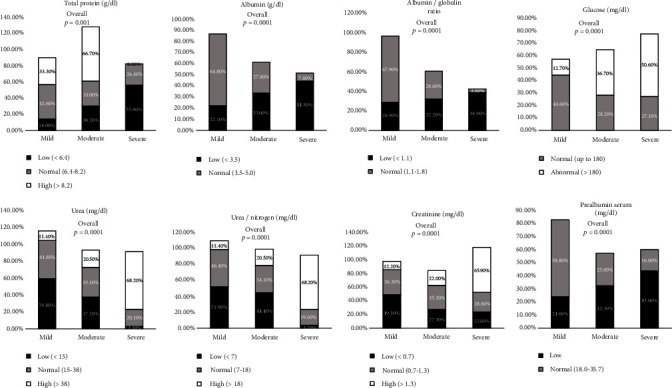
Comparison of baseline renal function test (RFT) marker levels between mild, moderate, and severe groups of COVID-19 patients on admission. Data are represented as *n* (%).

Figure 5 (Supplementary table S5) represents the frequency percentage of RFT and other biomarker levels on admission regarding COVID-19 severity.

Among the parameters, total protein (*p* < 0.001), albumin (*p* < 0.0001), albumin/globulin (A/G) ratio (*p* < 0.0001), and prealbumin (*p* < 0.0001) showed a significant decrease in the severe group when compared with the mild and moderate groups, whereas glucose (*p* < 0.0001), urea (*p* < 0.0001), urea/nitrogen (*p* < 0.0001), and creatinine (*p* < 0.0001) showed a significant increase in the severe group when compared with the moderate and mild groups. The percentage of total protein in the range of <6.4, 6.4-8.2, and >8.2 was 55.8%, 26.4%, and 0% in the severe group, whereas 30.2%, 31.0%, and 66.7% in the moderate group and 14.0%, 42.6%, and 33.3% in the mild group. The percentage of albumin in the range of >3.5 and 3.5-5.0 was 44.3% and 7.4% in the severe group, whereas 33.6% and 27.8% in the moderate group and 22.1% and 64.8% in the mild group. The A/G ratio in the range of <1.1 and 1.1-1.8 was 38.9% and 3.6% in the severe group, whereas 32.2% and 28.6% in the moderate group and 28.9% and 67.9% in the mild group. Glucose in the range of 180 and >180 was 27.1% and 50.6% in the severe group, whereas 28.2% and 36.7% in the moderate group and 44.6% and 12.7% in the mild group. The percentage of urea in the range of <15, 15-38, and >38 was 3.1%, 20.1%, and 68.2% in the severe group, whereas 37.5%, 35.1%, and 20.5% in the moderate group and 59.4%, 44.8%, and 11.4% in the mild group. The percentage of urea nitrogen in the range of <7, 7-18, and >18 was 3.7%, 19.6%, and 68.2% in the severe group, whereas 44.4%, 34.1%, and 20.5% in the moderate group and 51.9%, 46.4%, and 11.4% in the mild group. The percentage of creatinine in the range of <0.7, 0.7-1.3, and >1.3 was 23.6%, 28.6%, and 65.9% in the severe group, whereas 27.3%, 35.1%, and 22.0% in the moderate group and 49.1%, 36.3%, and 12.2% in the mild group. The percentage of prealbumin in the range of <18 and 18-35.7 was 43.7% and 16.3% in the severe group, whereas 32.3% and 25.0% in the moderate group and 24.0% and 58.8% in the mild group.

### 3.7. Risk Estimation by Multivariate Logistic Regression Analysis

Multivariate regression analysis was performed to obtain statistically significant independent determinants of COVID-19 illness severity in the final model (Table 3). We performed a multivariate analysis separately for haematological parameters, blood cell count index ratio, inflammatory markers, coagulation markers, LFT, RFT, and other biomarkers. In this analysis, WBC count-low (OR = 3.523, 95% CI = 1.182 to 10.502, *p* = 0.024), NLPR-high (OR = 4.135, 95% CI = 1.440 to 11.875, *p* = 0.008), neutrophils-high (OR = 6.246, 95% CI = 2.560 to 15.239, *p* = 0.0001), albumin (OR = 12.731, 95% CI = 1.568 to 103.4, *p* = 0.017), and urea-high (OR = 6.306, 95% CI = 2.241 to 17.749, *p* = 0.0001) were significantly associated with COVID-19 disease severity. A trend towards statistical significance was observed in WBC count-low (AOR = 4.034, 95% CI = 1.375 to 11.830, *p* = 0.011), neutrophils-high (AOR = 12.429, 95% CI = 5.662 to 27.282, *p* = 0.0001), albumin (AOR = 13.210, 95% CI = 1.617 to 107.9, *p* = 0.016), and urea-high (AOR = 7.120, 95% CI = 2.466 to 20.552, *p* = 0.0001) after correction with age, gender, and comorbidities.

The outcome model included WBC count, neutrophils, NLPR, albumin, and urea in the multivariate regression analysis (Table 4). Among these parameters, WBC count-low (AOR = 6.888, 95% CI = 1.590 to 29.839, *p* = 0.010), neutrophils-high (AOR = 5.912, 95% CI = 2.131 to 16.402, *p* = 0.001), and urea-high (AOR = 4.834, 95% CI = 1.988 to 11.755, *p* = 0.001) were significantly associated with COVID-19 severity after correcting with age, gender, and comorbidities.

### 3.8. Diagnostic Efficiency Using Combined ROC Curve for WBC, Neutrophils, and Urea

**Figure 6:**
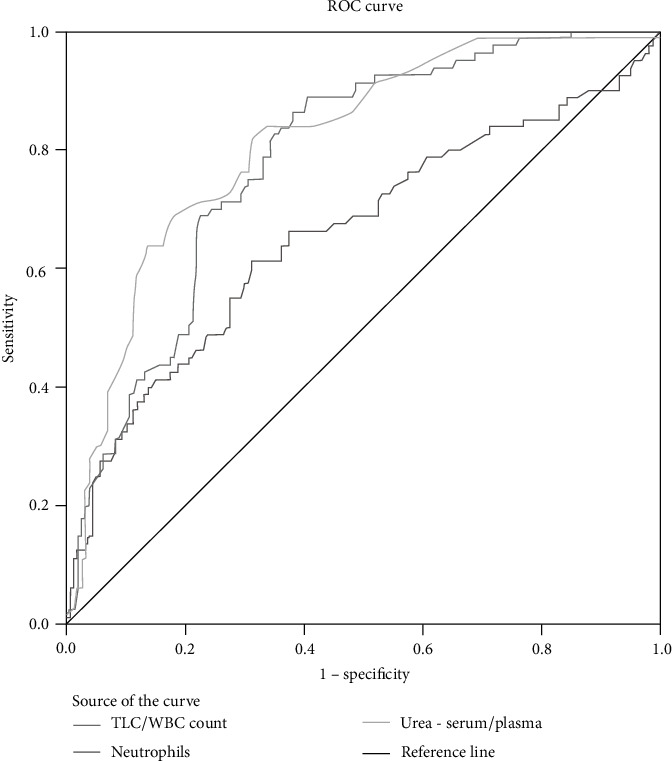
ROC curve analysis to predict the severity of COVID-19 for multivariate parameters WBC, neutrophils, and urea.

Figure 6 represents the ROC curve analysis for WBC, neutrophils, and urea, which has an AUC of 0.658, 0.783, and 0.814. This shows that WBC, neutrophils, and urea have a good diagnostic efficiency for predicting the severity of COVID-19 during admission.

## 4. Discussion

This retrospective study identified clinical markers for predicting the severity of COVID-19 on admission. Timely identification of predictive laboratory biomarkers could effectively personalise patient management. Multivariate logistic regression revealed that WBC count, neutrophils, and urea were independent prognostic factors for predicting disease severity, independent of age, gender, and comorbid factors.

A higher percentage of elderly patients were observed in the severe group than younger patients. The frequency of males was higher in all the groups than the females, consistent with previous reports [11, 12]. The apparent COVID-19 symptoms observed upon admission in severe patients were fever, increased pulse and respiratory rate, difficulty breathing, and dry cough. The most common comorbidities in severe COVID-19 patients were hypertension and diabetes mellitus. A decrease in Spo2 levels was observed in severe patients compared with the mild and moderate groups upon admission. In a previous study, vitals on admission, including pulse, respiratory rate, Spo2, comorbidities, older age, and male gender, are associated with COVID-19 severity [13].

In our study, the haematological parameters WBC count and neutrophils showed a significant (*p* = 0.0001) increase in the severe group compared with other groups. Our results were consistent with the previous study, which reported that neutrophil counts and TLC mean were higher in critically ill COVID-19 positive cases and 87.5% of critical patients with neutrophilia [14]. Furthermore, a decrease in eosinophil and lymphocyte count on admission is an important prognostic indicator of COVID-19 severity, consistent with the reported findings [15, 16].

Inflammation index markers derived from haematological parameters help assess COVID-19 severity [17, 18]. In our study, a significant (*p* = 0.0001) increase was observed in AISI, dNLR, MLR, NLPR, PLR, SII, and SIRI ratios in the severe group compared with the mild and moderate groups. In addition, previous studies have reported a significant increase of the above biomarkers in COVID-19 nonsurvivors compared with survivors [17, 19].

Inflammation and coagulation biomarkers like IL-6, D-dimer, ferritin, and LDH were significantly increased in the severe groups compared with the mild and moderate groups. This suggested that the inflammatory response and coagulation were more prominent in severe patients, which directly correlates with the survival of the patients. Our results were consistent with other studies, where it has been shown that elevated levels of IL-6 were observed in severe patients compared with nonsevere patients [20, 21]. D-dimer levels were elevated in severe patients on admission and might be a useful prognostic indicator in the early stages of the infection [22]. Several studies have shown that LDH and ferritin were higher in severe patients than in nonsevere patients [3].

Other significantly elevated biomarkers were ALP, AST, glucose, urea, urea nitrogen, and creatinine upon admission. Previous studies have reported elevated levels of AST and ALP in COVID-19 patients on admission ranging from 14% to 53%. A study on 701 patients revealed that high serum creatinine levels on admission correlated with severity due to significant abnormalities in the coagulation pathway [5]. A decrease in total protein, albumin, and A/G ratio was observed in the severe group compared with other groups. Several studies have shown a reduction in serum albumin concentration based on the severity of the disease [23, 24]. Decreased serum albumin concentrations were significantly associated with increased severity [24].

In multivariant analysis, the significant parameters were adjusted with age, gender, and comorbidities. WBC count, neutrophils, and urea independently predicted the severity of the COVID-19 infection. According to previous reports, the WBC count was significantly higher in nonsurvivors compared to survivors during COVID-19 infection and resulted in poor clinical outcomes [25, 26]. Several studies have shown the role of increased neutrophil count in determining the prognosis and severity of COVID-19 infection [25, 27]. In addition, previous studies have shown that COVID-19 affects the kidneys apart from the lungs due to ACE-2 receptors on their cellular surface [28–30]. In addition, studies have shown significantly elevated levels of serum urea in severe cases [28].

During COVID-19 infection, the virus infects the respiratory system, enters other cells, induces cytokine storm, and generates a series of immune reactions leading to increased WBC and neutrophil count. This cytokine storm leads to severe inflammation and direct or indirect activation of ACE-2 receptors in the kidney, leading to kidney damage [30].

Previous studies have shown that COVID-19 severity is associated with the host immune response system [31]. There is a significant change in the peripheral blood parameters related to immune pathogenesis of COVID-19 [31]. Among the peripheral blood parameters, WBC and neutrophils play a major role in the immune pathogenesis of COVID-19 [31, 32]. During COVID-19 infection, lymphocytes trigger the release of virus-related inflammatory factors, which leads to an increase in neutrophils in the blood [31, 32]. So impaired lymphocytes play a major role in releasing neutrophils during COVID-19 infection, which has a crucial role in producing inflammatory cytokines, leading to cytokine storm in COVID-19 [32].

This study has limitations: retrospective data and omission of several cases due to incomplete data sets. In addition, all the potential variables in their entirety were not evaluated. Finally, the sample size was limited to a single centre.

In conclusion, identifying a patient's severity in a shorter span at the time of hospital admission before the disease progression could be possible. In this study, we have assessed laboratory markers that can predict disease severity and outcome. Accordingly, leucocytosis, neutrophils, and urea elevation were associated with severe COVID-19 infection and were significant predictors of developing severe course of COVID-19 disease.

## Figures and Tables

**Table 1:** Blood cell count indexes and their formulas.

Blood cell count index	Formulas
AISI (aggregate index of systemic inflammation)	Neutrophil^∗^platelet count^∗^ monocyte to lymphocyte
dNLR (derived neutrophil-to-lymphocyte ratio)	Neutrophil/lymphocyte
MLR (monocyte-to-lymphocyte ratio)	Monocyte/lymphocyte
MPR (mean platelet volume to platelet ratio)	MPV/platelet count
NLPR (neutrophil-to-lymphocyte^∗^platelet ratio)	Neutrophil/lymphocyte^∗^platelet count
PLR (platelet-to-lymphocyte ratio)	Platelet count/lymphocyte
SII (systemic immune-inflammation index)	Neutrophil^∗^platelet-to-lymphocyte ratio
SIRI (systemic inflammation response index)	Neutrophil^∗^monocyte-to-lymphocyte ratio

**Table 2:** Baseline demographics and clinical characteristics between mild, moderate, and severe groups of COVID-19 patients on admission.

Demographic and clinical characteristics	Total (*n* = 350)			
	Mild (117)	Moderate (99)	Severe (134)	*p* value
Age				
18-44	64.3%	23.5%	12.2%	0.0001^∗∗∗∗^
45-59	30.5%	27.6%	41.9%	
≥60	15.6%	30.5%	53.9%	
Gender				
Male	60.7%	82.8%	77.6%	0.001^∗∗∗^
Female	39.3%	16.2%	21.6%	
Comorbidities				
Diabetes mellitus	24 (15.1%)	53 (33.3%)	82 (51.6%)	0.0001^∗∗∗∗^
Hypertension	24 (16%)	49 (32.7%)	77 (51.3%)	0.0001^∗∗∗∗^
CAD	5 (8.6%)	21 (36.2%)	32 (55.2%)	0.0001^∗∗∗∗^
CKD	0	6 (23.1%)	20 (76.9%)	0.0001^∗∗∗∗^
Others	26 (27.4%)	29 (30.5%)	40 (42.1%)	0.027^∗^
Clinical manifestations				
Fever	85 (34.8%)	73 (30%)	86 (35.2%)	0.204
Dry cough	62 (33.3%)	56 (30%)	68 (37%)	0.678
Sore throat	23(57.5%)	15 (37.5%)	2 (5%)	0.0001^∗∗∗∗^
Difficulty in breathing	21 (14.4%)	40 (27.4%)	85 (58.2%)	0.0001^∗∗∗∗^
Vitals on admission				
Pulse	86.94 ± 13.74	87.07 ± 12.75	98.14 ± 23.11	0.0001^∗∗∗∗^
Respiratory rate	21.31 ± 6.039	22.78 ± 4.3	27.98 ± 7.8	0.0001^∗∗∗∗^
Spo2	97.24 ± 2.207	95.96 ± 3.715	85.46 ± 16.84	0.0001^∗∗∗∗^
Survivors	117 (42.7%)	88 (32.1%)	69 (25.2%)	0.0001^∗∗∗∗^
Nonsurvivors	0	11 (14.5%)	65 (85.5%)	0.0001^∗∗∗∗^

Data are represented as *n* (%) or mean ± S.E. The asterisk (^∗^) indicates statistical significance.

**Table 3:** Risk estimation (odds ratio) of different parameters by multivariant logistic regression.

Parameter	Odds ratio (OR)	95% CI		*p* value	Adjusted odds ratio (AOR)	95% CI		*p* value
		Lower	Upper					
						Lower	Upper	
*Hematological parameters*								
WBC count-low	3.523	1.182	10.502	0.024	4.034	1.375	11.830	0.011^∗∗^
WBC count-high	1.894	1.007	3.564	0.048	1.914	1.006	3.639	0.048^∗^
NLPR-high	4.135	1.440	11.875	0.008				
Neutrophils-high	6.246	2.560	15.239	0.0001	12.429	5.662	27.282	0.0001^∗∗∗∗^
*Biochemical markers*								
Albumin	12.731	1.568	103.363	0.017	13.210	1.617	107.938	0.016^∗∗^
Urea-low	0.305	0.035	2.663	0.283	0.305	0.035	2.658	0.282
Urea-high	6.306	2.241	17.749	0.0001	7.120	2.466	20.552	0.0001^∗∗∗∗^

**Table 4:** Risk estimation (odds ratio) of different parameters by multivariant logistic regression.

Parameter	Odds ratio (OR)	95% CI		*p* value	Adjusted odds ratio (AOR)	95% CI		*p* value
		Lower	Upper					
						Lower	Upper	
WBC count-low	6.928	1.596	30.075	0.010	6.888	1.590	29.839	0.01^∗^
WBC count-high	1.381	0.513	3.722	0.523	1.338	0.495	3.619	0.566
Neutrophils-high	5.921	2.131	16.448	0.001	5.912	2.131	16.402	0.001^∗∗^
Urea-low	0.172	0.017	1.727	0.135	0.170	0.017	1.714	0.133
Urea-high	4.960	2.044	12.036	0.0001	4.834	1.988	11.755	0.001^∗∗^

## Data Availability

Request should be placed to the corresponding author.

## Abbreviations

COVID-19: Coronavirus disease 2019
AISI: Aggregate index of systemic inflammation
dNLR: Neutrophil-to-lymphocyte ratio
NLPR: Neutrophil-to-lymphocyte and platelet ratio
MLR: Monocyte-to-lymphocyte ratio
SII: Systemic immune-inflammation index
SIRI: Systemic inflammation response index
IL-6: Interleukin-6
LDH: Lactate dehydrogenase
PT: Prothrombin time
ALP: Alanine phosphatase
AST: Aspartate aminotransferase
ALT: Alanine amino transferase
MPR: Mean platelet volume to platelet ratio
GGTP: Gamma-glutamyl transferase
AOR: Adjusted odds ratio
CI: Confidence interval
OR: Odds ratio.

## Supplementary Materials

Supplementary Materials Table S1: comparison of baseline hematological parameters between mild, moderate, and severe groups of COVID-19 patients on admission. Table S2: comparison of baseline blood cell count derived inflammation index markers between mild, moderate, and severe groups of COVID-19 patients on admission. Table S3: comparison of baseline inflammation and coagulation marker levels between mild, moderate, and severe groups of COVID-19 patients on admission. Table S4: comparison of baseline liver function test (LFT) marker levels between mild, moderate, and severe groups of COVID-19 patients on admission. Table S5: comparison of baseline renal function test (RFT) marker levels between mild, moderate, and severe groups of COVID-19 patients on admission.
