# Supplementary material for: Diagnostic Values of Laboratory Biomarkers in Predicting a Severe Course of COVID-19 on Hospital Admission
Source: Biomed Res Int. 2022 Nov 7;2022:5644956. doi: 10.1155/2022/5644956 (PMC10027459; doi:10.1155/2022/5644956)
Supplement: Supplementary Materials — Table S1: comparison of baseline hematological parameters between mild, moderate, and severe groups of COVID-19 patients on admission. Table S2: comparison of baseline blood cell count derived inflammation index markers between mild, moderate, and severe groups of COVID-19 patients on admission. Table S3: comparison of baseline inflammation and coagulation marker levels between mild, moderate, and severe groups of COVID-19 patients on admission. Table S4: comparison of baseline liver function test (LFT) marker levels between mild, moderate, and severe groups of COVID-19 patients on admission. Table S5: comparison of baseline renal function test (RFT) marker levels between mild, moderate, and severe groups of COVID-19 patients on admission. [file 5644956.f1.docx]

**Table S1: Comparison of baseline hematological parameters between mild, moderate, and severe groups of COVID-19 patients on admission**

| **Parameters** | **Category/Reference values** | **Mild** | **Moderate** | **Severe** | **Chi-square** | **p-value** |
| --- | --- | --- | --- | --- | --- | --- |
| *Hemoglobin (g/dl)* | *Low (<11.0)* | 25.5% | 27.7% | 46.8% | 5.331 | 0.255 |
|  | *Normal (11.0-16.0)* | 35.9% | 28.9% | 35.2% |  |  |
|  | *High (>16.0)* | 30.0% | 10.0% | 60.0% |  |  |
|  |  |  |  |  |  |  |
| *Hematocrit (%)* | *Low (<34.0)* | 24.4% | 28.9% | 46.7% | 3.276 | 0.513 |
|  | *Normal (34.0-48.0)* | 35.1% | 28.2% | 36.7% |  |  |
|  | *High (>48.0)* | 43.5% | 26.1% | 30.4% |  |  |
|  |  |  |  |  |  |  |
| *RBC count (million/ul)* | *Low (<3.7)* | 20.0% | 30.0% | 50.0% | 6.264 | 0.180 |
|  | *Normal (3.7-5.6)* | 34.4% | 28.2% | 37.4% |  |  |
|  | *High (>5.6)* | 52.4% | 23.8% | 23.8% |  |  |
|  |  |  |  |  |  |  |
| *MCV (fl)* | *Low (<75)* | 45.0% | 25.0% | 30.0% | 8.716 | 0.069 |
|  | *Normal (75-95)* | 35.6% | 29.2% | 35.2% |  |  |
|  | *High (>95)* | 20.0% | 22.5% | 57.5% |  |  |
|  |  |  |  |  |  |  |
| *MCH (pg)* | *Low (<26)* | 43.6% | 27.3% | 29.1% | 5.918 | 0.205 |
|  | *Normal (26-32)* | 32.8% | 28.7% | 38.5% |  |  |
|  | *High (>32)* | 18.2% | 18.2% | 63.6% |  |  |
|  |  |  |  |  |  |  |
| *MCHC (g/dl)* | *Low (<31)* | 32.8% | 23.9% | 43.3% | 1.294 | 0.524 |
|  | *Normal (31-36)* | 34.6% | 29.3% | 36.2% |  |  |
|  |  |  |  |  |  |  |
| *RDW-CV (%)* | *Normal (11-16)* | 34.9% | 29.1% | 36.0% | 2.850 | 0.240 |
|  | *High (>16)* | 28.9% | 21.1% | 50.0% |  |  |
|  |  |  |  |  |  |  |
| *Platelet count (10^3^/mm^3^)* | *Low (<140)* | 18.2% | 27.3% | 54.5% | 2.339 | 0.674 |
|  | *Normal (140-440)* | 34.7% | 28.5% | 36.8% |  |  |
|  | *High (>440)* | 36.4% | 18.2% | 45.5% |  |  |

**Table S1: continued**

| **Parameters** | **Category/Reference values** | **Mild** | **Moderate** | **Severe** | **Chi-square** | **p-value** |
| --- | --- | --- | --- | --- | --- | --- |
| *MPV (fl)* | *Normal (6.5-12)* | 34.1% | 28.5% | 37.4% | 1.068 | 0.586 |
|  | *High (>16.0)* | 37.5% | 12.5% | 50.0% |  |  |
|  |  |  |  |  |  |  |
| *WBC count (10^3^/mm^3^)* | *Low (<4)* | 37.9% | 24.1% | 37.9% | 33.996 | **0.0001^****^** |
|  | *Normal (4-11)* | 40.3% | 31.8% | 28.0% |  |  |
|  | *High (>11)* | 15.1% | 19.2% | 65.8% |  |  |
|  |  |  |  |  |  |  |
| *Neutrophils (%)* | *Low (<40)* | 77.8% | 11.1% | 11.1% | 108.999 | **0.0001^****^** |
|  | *Normal (40-75)* | 62.0% | 27.1% | 10.9% |  |  |
|  | *High (>75)* | 11.4% | 29.7% | 58.9% |  |  |
|  |  |  |  |  |  |  |
| *Lymphocytes (%)* | *Low (<20)* | 19.3% | 28.8% | 51.9% | 77.875 | **0.0001^****^** |
|  | *Normal (20-45)* | 64.4% | 27.6% | 8.0% |  |  |
|  | *High (>45)* | 71.4% | 21.4% | 7.1% |  |  |
|  |  |  |  |  |  |  |
| *Eosinophils (%)* | *Low (<1)* | 25.0% | 31.0% | 44.0% | 45.897 | **0.0001^****^** |
|  | *Normal (1-6)* | 68.3% | 16.7% | 15.0% |  |  |
|  | *High (>6)* | 80.0% | 20.0% | 0.0% |  |  |
|  |  |  |  |  |  |  |
| *Monocytes (%)* | *Low (<1)* | 66.7% | 0.0% | 33.3% | 6.148 | 0.188 |
|  | *Normal (1-10)* | 32.5% | 28.4% | 39.1% |  |  |
|  | *High (>10)* | 52.4% | 28.6% | 19.0% |  |  |
|  |  |  |  |  |  |  |
| *Basophils (%)* | *Normal (0-1)* | 33.6% | 29.1% | 37.4% | 0.808 | 0.668 |
|  | *High (>1)* | 50.0% | 16.7% | 33.3% |  |  |

**Data are represented as n (%). The asterisk (*) indicates statistical significance**

**Table S2: Comparison of baseline blood cell count derived inflammation index markers between mild, moderate, and severe groups of COVID-19 patients on admission**

| **Parameters** | **Category** | **Mild** | **Moderate** | **Severe** | **Chi-square** | **p-value** |
| --- | --- | --- | --- | --- | --- | --- |
| *AISI* | *Low (<280)* | 50.0% | 0.0% | 50.0% | 61.679 | **0.0001^****^** |
|  | *Normal (280-7333)* | 50.6% | 28.2% | 21.3% |  |  |
|  | *High (>7333)* | 12.6% | 28.9% | 58.5% |  |  |
|  |  |  |  |  |  |  |
| *dNLR* | *Low (<2)* | 72.2% | 25.0% | 2.8% | 50.883 | **0.0001^****^** |
|  | *Normal (2-1.6)* | 66.7% | 22.2% | 11.1% |  |  |
|  | *High (>1.6)* | 25.2% | 29.2% | 45.6% |  |  |
|  |  |  |  |  |  |  |
| *MLR* | *Normal (0.05-0.2)* | 56.1% | 29.8% | 14.0% | 20.528 | **0.0001^****^** |
|  | *High (>0.2)* | 28.8% | 28.4% | 42.8% |  |  |
|  |  |  |  |  |  |  |
| *MPR* | *Low (<0.046)* | 34.1% | 26.8% | 39.0% | 0.243 | 0.993 |
|  | *Normal (0.046-0.027)* | 35.0% | 28.5% | 36.5% |  |  |
|  | *High (>0.027)* | 32.8% | 28.4% | 38.8% |  |  |
|  |  |  |  |  |  |  |
| *NLPR* | *Low (<0.014)* | 90.9% | 9.1% | 0.0% | 93.763 | **0.0001^****^** |
|  | *Normal (0.014-0.037)* | 65.2% | 27.2% | 7.6% |  |  |
|  | *High (>0.037)* | 17.6% | 29.5% | 52.9% |  |  |
|  |  |  |  |  |  |  |
| *PLR* | *Low (<7)* | 57.4% | 27.9% | 14.7% | 58.496 | **0.0001^****^** |
|  | *Normal (7-9.7)* | 60.0% | 28.9% | 11.1% |  |  |
|  | *High (>9.7)* | 20.5% | 28.0% | 51.5% |  |  |
|  |  |  |  |  |  |  |
| *SII* | *Low (<280)* | 58.1% | 29.0% | 12.9% | 80.813 | **0.0001^****^** |
|  | *Normal (280-733)* | 63.3% | 25.6% | 11.1% |  |  |
|  | *High (>733)* | 16.7% | 29.2% | 54.2% |  |  |
|  |  |  |  |  |  |  |
| *SIRI* | *Low (<2)* | 50.0% | 0.0% | 50.0% | 51.603 | **0.0001^****^** |
|  | *Normal (2-16.6)* | 59.6% | 27.3% | 13.1% |  |  |
|  | *High (>16.6)* | 22.2% | 28.8% | 49.1% |  |  |

**Data are represented as n (%). The asterisk (*) indicates statistical significance**

**Table S3: Comparison of baseline Inflammation and coagulation marker levels between mild, moderate, and severe groups of COVID-19 patients on admission**

| **Parameters** | **Category** | **Mild** | **Moderate** | **Severe** | **Chi-square** | **p-value** |
| --- | --- | --- | --- | --- | --- | --- |
| *IL-6 (pg/ml)* | *Low (<5.3)* | 70.4% | 25.9% | 3.7% | 31.382 | **0.0001^****^** |
|  | *Normal (5.3-7.5)* | 63.6% | 27.3% | 9.1% |  |  |
|  | *High (>7.5)* | 22.9% | 37.5% | 39.6% |  |  |
|  |  |  |  |  |  |  |
| *D-Dimer (ng/ml)* | *Normal (<500)* | 47.5% | 27.7% | 24.8% | 8.787 | **0.012^**^** |
|  | *Abnormal (>500)* | 28.8% | 33.6% | 37.6% |  |  |
|  |  |  |  |  |  |  |
| *Prothrombin time (secs)* | *Normal (11-14)* | 50.0% | 19.4% | 30.6% | 9.662 | **0.047^*^** |
|  | *High (>14)* | 23.4% | 42.6% | 34.0% |  |  |
|  |  |  |  |  |  |  |
| *Activated prothrombin time (sec)* | *Low (<28)* | 43.8% | 25.0% | 31.3% | 1.558 | 0.816 |
|  | *Normal (28-40)* | 36.1% | 36.1% | 27.9% |  |  |
|  | *High (>40)* | 25.0% | 25.0% | 50.0% |  |  |
|  |  |  |  |  |  |  |
| *Ferritin (ng/ml)* | *Low (<23.9)* | 85.7% | 14.3% | 0.0% | 32.689 | **0.0001^****^** |
|  | *Normal (23.9-336.2)* | 47.9% | 34.4% | 17.7% |  |  |
|  | *High (>336.2)* | 16.3% | 37.5% | 46.3% |  |  |
|  |  |  |  |  |  |  |
| *LDH (U/L)* | *Normal (85-227)* | 65.3% | 18.4% | 16.3% | 27.757 | **0.0001^****^** |
|  | *High (>227)* | 25.3% | 35.7% | 39.0% |  |  |

**Data are represented as n (%). The asterisk (*) indicates statistical significance**

**Table S4: Comparison of baseline Liver function test (LFT) marker levels between mild, moderate, and severe groups of COVID-19 patients on admission**

| **Parameters** | **Category** | **Mild** | **Moderate** | **Severe** | **Chi-square** | **p-value** |
| --- | --- | --- | --- | --- | --- | --- |
| *Bilirubin-total (mg/dl)* | *Normal (<=1.0)* | 37.3% | 32.3% | 30.4% | 4.814 | 0.090 |
|  | *Abnormal (>1.0)* | 15.8% | 31.6% | 52.6% |  |  |
|  |  |  |  |  |  |  |
| *Bilirubin-conjugated (mg/dl)* | *Normal (0-0.3)* | 45.1% | 32.7% | 22.1% | 3.051 | 0.218 |
|  | *Abnormal (>0.3)* | 18.2% | 45.5% | 36.4% |  |  |
|  |  |  |  |  |  |  |
| *Bilirubin-Unconjugated (mg/dl)* | *Normal (0-0.7)* | 44.1% | 33.1% | 22.9% | 1.683 | 0.431 |
|  | *Abnormal (>0.7)* | 20.0% | 60.0% | 20.0% |  |  |
|  |  |  |  |  |  |  |
| *ALP (U/L)* | *Low (<46)* | 22.2% | 22.2% | 55.6% | 13.408 | **0.009^***^** |
|  | *Normal (46-116)* | 39.2% | 33.1% | 27.7% |  |  |
|  | *High (>116)* | 9.5% | 28.6% | 61.9% |  |  |
|  |  |  |  |  |  |  |
| *ALT (U/L)* | *Low (<16)* | 25.0% | 25.0% | 50.0% | 1.129 | 0.890 |
|  | *Normal (16-63)* | 34.7% | 32.6% | 32.6% |  |  |
|  | *High (>63)* | 37.0% | 29.6% | 33.3% |  |  |
|  |  |  |  |  |  |  |
| *AST (U/L)* | *Low (<15)* | 86.7% | 6.7% | 6.7% | 18.351 | **0.001^****^** |
|  | *Normal (15-37)* | 37.5% | 34.1% | 28.4% |  |  |
|  | *High (>37)* | 26.8% | 35.7% | 37.5% |  |  |
|  |  |  |  |  |  |  |
| *GGTP (U/L)* | *Normal (15-85)* | 45.4% | 33.0% | 21.6% | 2.793 | 0.593 |
|  | *High (>85)* | 32.0% | 40.0% | 28.0% |  |  |
|  |  |  |  |  |  |  |
| *Amylase (U/L)* | *Normal (25-115)* | 21.8% | 30.9% | 47.3% | 1.338 | 0.855 |
|  | *High (>115)* | 25.0% | 25.0% | 50.0% |  |  |

**Data are represented as n (%). The asterisk (*) indicates statistical significance**

**Table S5: Comparison of baseline Renal function test (RFT) marker levels between mild, moderate, and severe groups of COVID-19 patients on admission**

| **Parameters** | **Category/Reference value** | **Mild** | **Moderate** | **Severe** | **Chi-square** | **p-value** |
| --- | --- | --- | --- | --- | --- | --- |
| *Total protein (g/dl)* | *Low (<6.4)* | 14.0% | 30.2% | 55.8% | 18.160 | **0.001^***^** |
|  | *Normal (6.4-8.2)* | 42.6% | 31.0% | 26.4% |  |  |
|  | *High (>8.2)* | 33.3% | 66.7% | 0.0% |  |  |
|  |  |  |  |  |  |  |
| *Albumin (g/dl)* | *Low (>3.5)* | 22.1% | 33.6% | 44.3% | 36.397 | **0.0001^****^** |
|  | *Normal (3.5-5.0)* | 64.8% | 27.8% | 7.4% |  |  |
|  |  |  |  |  |  |  |
| *Globulin (g/dl)* | *Normal (1.8-3.6)* | 42.3% | 28.2% | 29.6% | 4.862 | 0.302 |
|  | *High (>3.6)* | 30.2% | 34.0% | 35.8% |  |  |
|  |  |  |  |  |  |  |
| *Albumin/Globulin ratio* | *Low (<1.1)* | 28.9% | 32.2% | 38.9% | 19.171 | **0.0001^****^** |
|  | *Normal (1.1-1.8)* | 67.9% | 28.6% | 3.6% |  |  |
|  |  |  |  |  |  |  |
| *Glucose (mg/dl)* | *Normal (up to 180)* | 44.6% | 28.2% | 27.1% | 26.115 | **0.0001^****^** |
|  | *Abnormal (>180)* | 12.7% | 36.7% | 50.6% |  |  |
|  |  |  |  |  |  |  |
| *Urea (mg/dl)* | *Low (<15)* | 59.4% | 37.5% | 3.1% | 73.352 | **0.0001^****^** |
|  | *Normal (15-38)* | 44.8% | 35.1% | 20.1% |  |  |
|  | *High (>38)* | 11.4% | 20.5% | 68.2% |  |  |
|  |  |  |  |  |  |  |
| *Urea/Nitrogen (mg/dl)* | *Low (<7)* | 51.9% | 44.4% | 3.7% | 71.595 | **0.0001^****^** |
|  | *Normal (7-18)* | 46.4% | 34.1% | 19.6% |  |  |
|  | *High (>18)* | 11.4% | 20.5% | 68.2% |  |  |
|  |  |  |  |  |  |  |
| *Creatinine (mg/dl)* | *Low (<0.7)* | 49.1% | 27.3% | 23.6% | 27.135 | **0.0001^****^** |
|  | *Normal (0.7-1.3)* | 36.3% | 35.1% | 28.6% |  |  |
|  | *High (>1.3)* | 12.2% | 22.0% | 65.9% |  |  |
|  |  |  |  |  |  |  |
| *Prealbumin serum (mg/dl)* | *Low* | 24.0% | 32.3% | 43.7% | 31.873 | **0.0001^****^** |
|  | *Normal (18.0-35.7)* | 58.8% | 25.0% | 16.3% |  |  |
|  |  |  |  |  |  |  |
| *Uric acid (mg/dl)* | *Normal (3.5-7.2)* | 19.0% | 33.3% | 47.6% | 5.795 | 0.215 |
|  | *Low* | 26.3% | 15.8% | 57.9% |  |  |
|  |  |  |  |  |  |  |
| *Cholesterol (mg/dl)* | *Normal (200-240)* | 50.0% | 0.0% | 50.0% | 1.788 | 0.409 |
|  | *Low* | 16.7% | 28.3% | 55.0% |  |  |

**Data are represented as n (%). The asterisk (*) indicates statistical significance**
